# Salivary Microbiota for Gastric Cancer Prediction: An Exploratory Study

**DOI:** 10.3389/fcimb.2021.640309

**Published:** 2021-03-10

**Authors:** Kun Huang, Xuefeng Gao, Lili Wu, Bin Yan, Zikai Wang, Xiaomei Zhang, Lihua Peng, Jiufei Yu, Gang Sun, Yunsheng Yang

**Affiliations:** ^1^Department of Gastroenterology and Hepatology, The First Medical Center, Chinese PLA General Hospital, Medical School of Chinese PLA, Beijing, China; ^2^Department of Gastroenterology, Civil Aviation General Hospital, Beijing, China; ^3^Central Laboratory, Shenzhen Key Laboratory of Precision Medicine for Hematological Malignancies, Shenzhen University General Hospital, Shenzhen, China; ^4^Department of Gastroenterology, The Second Medical Center, Chinese PLA General Hospital, Beijing, China

**Keywords:** gastric cancer, precancerous lesions, salivary microbiota, 16S rRNA, high-throughput sequencing

## Abstract

To characterize the salivary microbiota in patients at different progressive histological stages of gastric carcinogenesis and identify microbial markers for detecting gastric cancer, two hundred and ninety-three patients were grouped into superficial gastritis (SG; n = 101), atrophic gastritis (AG; n = 93), and gastric cancer (GC; n = 99) according to their histology. 16S rRNA gene sequencing was used to access the salivary microbiota profile. A random forest model was constructed to classify gastric histological types based on the salivary microbiota compositions. A distinct salivary microbiota was observed in patients with GC when comparing with SG and AG, which was featured by an enrichment of putative proinflammatory taxa including *Corynebacterium* and *Streptococcus*. Among the significantly decreased oral bacteria in GC patients including *Haemophilus*, *Neisseria*, *Parvimonas*, *Peptostreptococcus*, *Porphyromonas*, and *Prevotella*, *Haemophilus*, and *Neisseria* are known to reduce nitrite, which may consequently result in an accumulation of carcinogenic N-nitroso compounds. We found that GC can be distinguished accurately from patients with AG and SG (AUC = 0.91) by the random forest model based on the salivary microbiota profiles, and taxa belonging to *unclassified Streptophyta* and *Streptococcus* have potential as diagnostic biomarkers for GC. Remarkable changes in the salivary microbiota functions were also detected across three histological types, and the upregulation in the isoleucine and valine is in line with a higher level of these amino acids in the gastric tumor tissues that reported by other independent studies. Conclusively, bacteria in the oral cavity may contribute gastric cancer and become new diagnostic biomarkers for GC, but further evaluation against independent clinical cohorts is required. The potential mechanisms of salivary microbiota in participating the pathogenesis of GC may include an accumulation of proinflammatory bacteria and a decline in those reducing carcinogenic N-nitroso compounds.

## Introduction

Gastric cancer (GC) constitutes the third highest cause of cancer mortality worldwide (Bray et al., 2018), and the 5-year survival rates are 27.4 and 32% in China and the USA, respectively. The occurrence and development of gastric carcinogenesis is a complex pathogenic process involving multiple factors, multi-stage changes and polygenic alterations (Massarrat and Stolte, 2014; Goral, 2016). A late-stage presentation is common in most GC cases, because symptoms in early stages of the disease are usually vague and non-specific. As early detection leads to better outcomes, there is a critical need for new avenues of prevention, risk stratification, and early detection for GC.

Microbes in the upper digestive tract have been shown to facilitate carcinogenesis by contributing inflammatory processes *via* activation of Toll-like receptors pathway (Kauppila and Selander, 2014), or protect against carcinogenesis by providing barriers to pathogen invasion (Yang et al., 2014). Chronic infection with *Helicobacter pylori* is a well-established risk factor for gastric carcinogenesis. Lines of evidence demonstrated that the process of Correa’s cascade of gastric carcinogenesis initiated by *H. pylori* involves multiple virulence factors, host genetic make-up, and nutritional factors (Warren and Marshall, 1983; Polk and Peek, 2010; Engstrand and Lindberg, 2013; Plummer et al., 2015). Nevertheless, only about 3% of those infected with *H. pylori* will eventually develop into gastric cancer, and the eradication of *H. pylori* does not completely prevent the occurrence of GC (Peek and Crabtree, 2006). These lines of evidence suggest that non-*H. pylori* microorganisms colonizing the stomach may represent an additional modifier of gastric cancer risk (Sung et al., 2020). The enrichment of some bacteria in the gastric mucosa has been associated with the progression of gastric cancer, including *Peptostreptococcus stomatis*, *Streptococcus anginosus*, *Parvimonas micra*, *Slackia exigua*, and *Dialister pneumosintes* (Coker et al., 2018). Our recent study suggested that a reduction of nitrite-oxidizing Nitrospirae taxa in the gastric mucosa may contribute to gastric neoplastic progression *via* nitrate accumulation (Wang et al., 2020).

Most of the microbial sources in the stomach are believed from the external environment. The oral cavity contains a large number of microorganisms, including bacteria, viruses, fungi, mycoplasma, and chlamydia (Aas et al., 2005; Wade, 2013; He et al., 2015). The oral microbiota can enter the downstream digestive tract from the oral cavity through saliva and can also migrate to various parts of the body to cause infections and local inflammatory reactions in corresponding sites (Han and Wang, 2013), oral microbes are closely correlated with several systemic diseases such as the oral tumors, type 2 diabetes, cardiovascular disease, urinary systemic diseases and rheumatoid arthritis (Seymour, 2010; Ahn et al., 2012; Salazar et al., 2012; Whitmore and Lamont, 2014; Gao et al., 2018). Recently, oral microbiota has been suggested to play a role in the etiology of esophageal cancer, colorectal cancer (CRC), and pancreatic cancer (Michaud and Izard, 2014; Peters et al., 2017; Flemer et al., 2018). Interestingly, a higher incidence of GC was found among people with worse oral hygiene (Watabe et al.), indicating a potential link between the oral microbiota and the occurrence/development of gastric cancer. In this study, we characterized the microbial compositional and ecological changes in salivary microbiota of patients with GC and non-malignant gastric lesions including superficial gastritis (SG) and atrophic gastritis (AG). We demonstrated the possibility of using salivary microbes as biomarkers GC detection, and explored the potential mechanisms of oral microbiota in the pathogenesis of GC.

## Materials and Methods

### Participants

Two hundred and ninety-three patients who received an endoscopic examination in the Chinese PLA General Hospital and Civil Aviation General Hospital were enrolled in this study. The study cohort was recruited from October 2017 to October 2019. The inclusion criteria were: (1) adult male or female; (2) Han nationality from northern China; (3) able and willing to provide signed and dated informed consent; (4) able and willing to provide salivary samples. The exclusion criteria were: (1) taking antibiotics, proton pump inhibitors (PPIs), probiotics, prebiotics, chemotherapeutic drugs, and any other drugs affecting oral microbiota within the last month; (2) diagnosed with acute or chronic pulmonary, cardiovascular, hepatic, or renal disorders; (3) positive test for human immunodeficiency virus, hepatitis B or C virus; (4) a history of major surgery; and (5) women who were pregnant or lactating.

Data collection was conducted for all subjects, including demographics, medical history, drugs, and hematology tests.

### Endoscopic and Histologic Examination

Patients’ diagnostic evaluation was based on the endoscopic and histological examination. SG was confirmed according to the infiltrating depth and density of chronic inflammatory cells in the mucosa, without the reduction of proper gastric glands at each biopsy site. If the gastric mucosa in the antrum and the body were atrophied and thinned, the submucosal vessels could be well visualized under the gastroscopy; in the meantime, the proper gastric glands reduced at each biopsy site, it was defined as AG. IM was defined as the replacement of gastric mucosal epithelial cells by intestinal epithelial cells at each biopsy site. GC was confirmed by the histological examination; according to WHO gastric adenocarcinoma grading criteria, it was divided into well differentiated, moderately differentiated, and poorly differentiated (Jean-François, 2011).

### Sample Collection

Salivary sample collection and preparation were carried out in accordance with previously published consensus (Shi et al., 2019). All the subjects were fasting and did not brush the teeth in the morning. Thirty minutes before sampling, subjects were asked to rinse the mouth with water, and then 1 ml saliva was collected in a sterilized tube containing 1.0 ml RNAlater (Life Technologies, USA), transferred to the laboratory and stored at room temperature until DNA extraction.

### DNA Extraction and 16S rRNA Gene Amplicons

To evaluate the salivary bacterial diversity, high-throughput sequencing of the 16S rRNA was performed. Bacterial genomic DNA of the saliva was isolated using the QIAamp DNA Mini Kit (QIAGEN, Valencia, CA, USA) combined with the bead-beating method. The DNA concentrations of each sample were adjusted to 50 ng/μl and stored at −80°C for sequencing. The hypervariable V3–V4 region of the 16S rRNA gene was amplified using the universal primers (515F, GTGCCAGCMGCCGCGGTAA and 806R, GGACTACHVGGGTWTCTAAT) with a 6-bp barcode. All PCR reactions (including denaturation, annealing and elongation) were carried out with Phusion ^®^ High-Fidelity PCR Master Mix (New England Biolabs). The single amplifications were performed in 25 µl reactions with 50 ng template DNA. Normalized equimolar concentrations of PCR products were pooled and sequenced using the Illumina MiSeq PE300 platform (Illumina, San Diego, CA, United States) at Shenzhen Decipher Biotechnology Laboratory.

We employed the QIIME 2 (Bolyen et al., 2019) *dada2 denoise-paired* method to denoise, dereplicate, and filter chimeras from the sequence data. For taxonomic classification, we trained a Naive Bayes classifier on the 16S rRNA V3–V4 regions with *q2-feature-classifier* method (**Supplementary File S1**). The metagenome functions of the salivary microbiota were predicted through PICRUSt2 on the basis of 16S rRNA gene sequencing profiles (Douglas et al., 2020).

### Statistical and Bioinformatic Analyses

The baseline continuous data were presented by mean ± standard deviation (SD) and analyzed by independent *t* test or non-parametric rank test. The categorical data were described in percentages and compared by χ^2^ test or Fisher’s exact test. All tests for significance were two-sided, and *P <*0.05 was considered significant.

Calypso (version 8.84) was used to conduct statistical analysis of the microbiota compositional data. The read counts were normalized with total sum normalization, and taxa having less than 0.02% relative abundance across all samples were excluded from the following analysis. The Amplicon sequence variant (ASV) counts were normalized with total-sum scaling (TSS) followed by cumulative-sum scaling (CSS). The alpha diversity of the salivary microbiota was measured by Shannon’s index and Chao1 index. The relative abundances of taxa were log2 transformed to account for the non-normality. Principal coordinate analysis (PCoA) based on unweighted and weighted UniFrac distance matrices were employed to stratify samples and identify group level clusters, and the corresponding statistical significance was assessed using Permutational multivariate analysis of variance (PERMANOVA). Anosim was applied to compare the intra-group distances with between-group distances. Kruskal–Wallis test was used to detect significant differences in the alpha diversity, abundances of taxa, and metabolic pathways across the histological stages, which was followed by Wilcoxon rank-sum test confirming the significant differences between each two groups. Benjamini–Hochberg (BH) procedure was applied to control the false discovery rate. The Linear discriminant analysis Effect Size (LEfSe) (Segata et al., 2011) was applied to identify the features (ASV or functions) most likely to explain differences in the salivary microbiota between histological types. Spearman correlation networks were constructed based on the top 30 most abundant genera and edges of correlations with Holm-corrected *P <*0.05 were shown.

The random forest (RF) model was built through the caret R package. Five-fold cross-validation and area under the receiver operating characteristic (ROC) curve (AUC) were used to evaluate the prediction performance of the model and was implemented using pROC R package. The RF disease classifier using oral bacterial abundances at the genus level was constructed with 60% randomly selected samples as the training set and tuneLength = 4. The R code and taxa abundance table used for constructing the random forest model are provided in the **Supplementary Code 1**.

## Results

### Demographic Characteristics of the Patient Cohort

After a standardized endoscopic procedure and histopathological evaluation, a total of 101 SG, 93 AG (21 without IM, 72 with IM), and 99 GC subjects were enrolled. The gender and age were matched among the four groups (*P* = 0.9152 and *P* = 0.3582, respectively). There were also no significant differences in body mass index (BMI), socioeconomic, medical history (including periodontosis), or lifestyle characteristics (smoking and drinking status) among the four groups (**Table 1**).

**Table 1:** Distribution of demographic characteristics among SG, AG, and GC.

Histological types	SG	AG		GC	P-value
		Without IM	With IM		
**n**	101	21	72	99	
**Gender (male, female)**	(51, 50)	(10, 11)	(39, 33)	(64, 35)	0.9152
**Age**	48.2 ± 10.2	49.9 ± 12.5	48.5 ± 11.7	49.6 ± 8.8	0.3582
**BMI**	23.5 ± 2.4	22.7 ± 3.4	22.8 ± 4.5	21.4 ± 2.2	0.8062
**Periodontosis**	15	13	21	42	0.0951
**Smoking**	34	10	29	40	0.2482
**Drinking**	42	14	38	51	0.8502

### Salivary Microbiota Changes Are Associated With Gastric Neoplastic Progression

**Figure 1:**
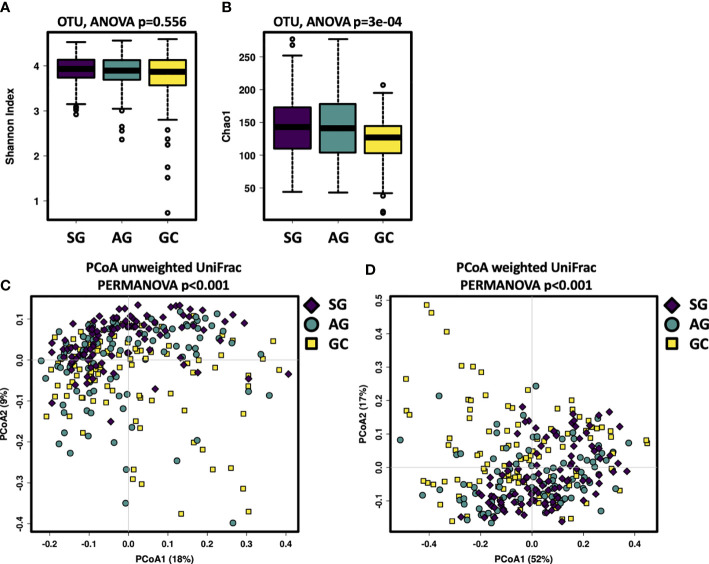
The salivary microbiota biodiversity in patients with malignant and non-malignant gastric lesions. The alpha diversity of salivary microbiota was measured at the ASV level by using **(A)** Shannon index, **(B)** Chao1. Significance was determined by using Kruskal–Wallis rank sum test, and Wilcoxon rank-sum tests for each of the two groups. PCoA based on **(C)** unweighted UniFrac distance matrix, and **(D)** weighted UniFrac distance matrix revealed distinct clustering of GC samples.

This study assessed the salivary microbiota by sequence analysis of the 16S ribosomal RNA gene. A total of 14,989,371 raw reads were obtained after quality filtering, with an average of 51,158 for each sample. The refined reads were clustered into 1,275 ASVs. The salivary microbiota alpha diversity was significantly lower in GC than that of SG and AG (**Figures 1A, B**). Beta analysis with PCoA showed that the cluster of GC samples could be separated from SG and AG (**Figures 1C, D**). Regarding AG, the alpha and beta diversities in the salivary microbiota in patients with and without intestinal metaplasia were not distinguishable (**Figure S1**). For GC patients with different histological grades (well differentiated, moderately differentiated, and poorly differentiated), there was no significant difference in the biodiversity among in the salivary microbiota (**Figure S2**).

**Figure 2:**
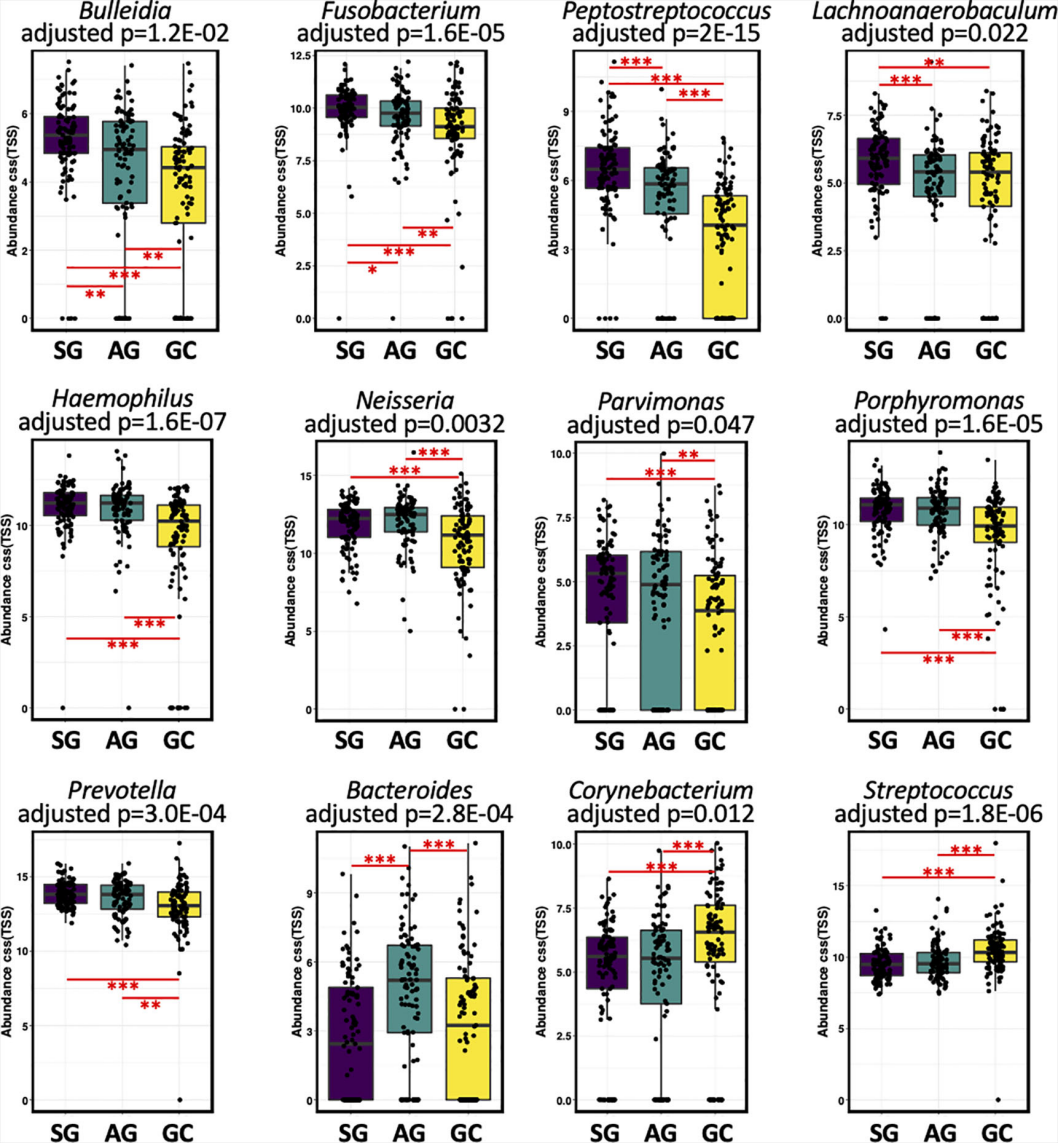
The salivary microbiota composition in patients with malignant and non-malignant gastric lesions. The salivary microbiota composition in patients with malignant and non-malignant gastric lesions. Relative abundance of salivary taxa of the three groups were compared at the genus level. Significance was determined by using Kruskal-Wallis rank sum test with BH-adjusted *P* < 0.001, and Wilcoxon rank-sum tests for each of the two groups with *BH-adjusted *P* < 0.05, **BH-adjusted *P* < 0.01, and ***BH-adjusted *P* < 0.001.

Compositionally, the most abundant phyla in the salivary microbiota are *Bacteroidetes, Protobacteria, Firmicutes, Fusobacteria*, and *Acinobacteria*, which account for more than 94% of the bacterial community for each histological stage of GC (**Figure S3A**). Patients with GC had a higher relative abundance of Cyanobacteria (**Table S1**). At the genus level, *Prevotella*, *Neisseria*, *Veillonella*, *Haemophilus*, *Porphyromonas*, *Streptococcus*, *Fusobacterium*, and *Rothia* constitute more than 70% of the salivary microbiota for each histological stage of GC (**Figure S3B**). The levels of *Anaerovorax, Bulleidia, unclassified F16*, and *Peptostreptococcus* gradually decreased from SG through AG to GC (**Figure 2**; **Table S1**), indicating a negative association of these bacteria with GC development. The genera *Streptococcus* and *unclassified Streptophyta* were significantly higher in GC, whereas *Fusobacterium*, *Haemophilus*, *Neisseria*, *Parvimonas*, *Peptostreptococcus*, *Porphyromonas*, and *Prevotella* were less abundant in GC. In addition, *Bacteroides* genus was found particularly more abundant in the patients with AG.

**Figure 3:**
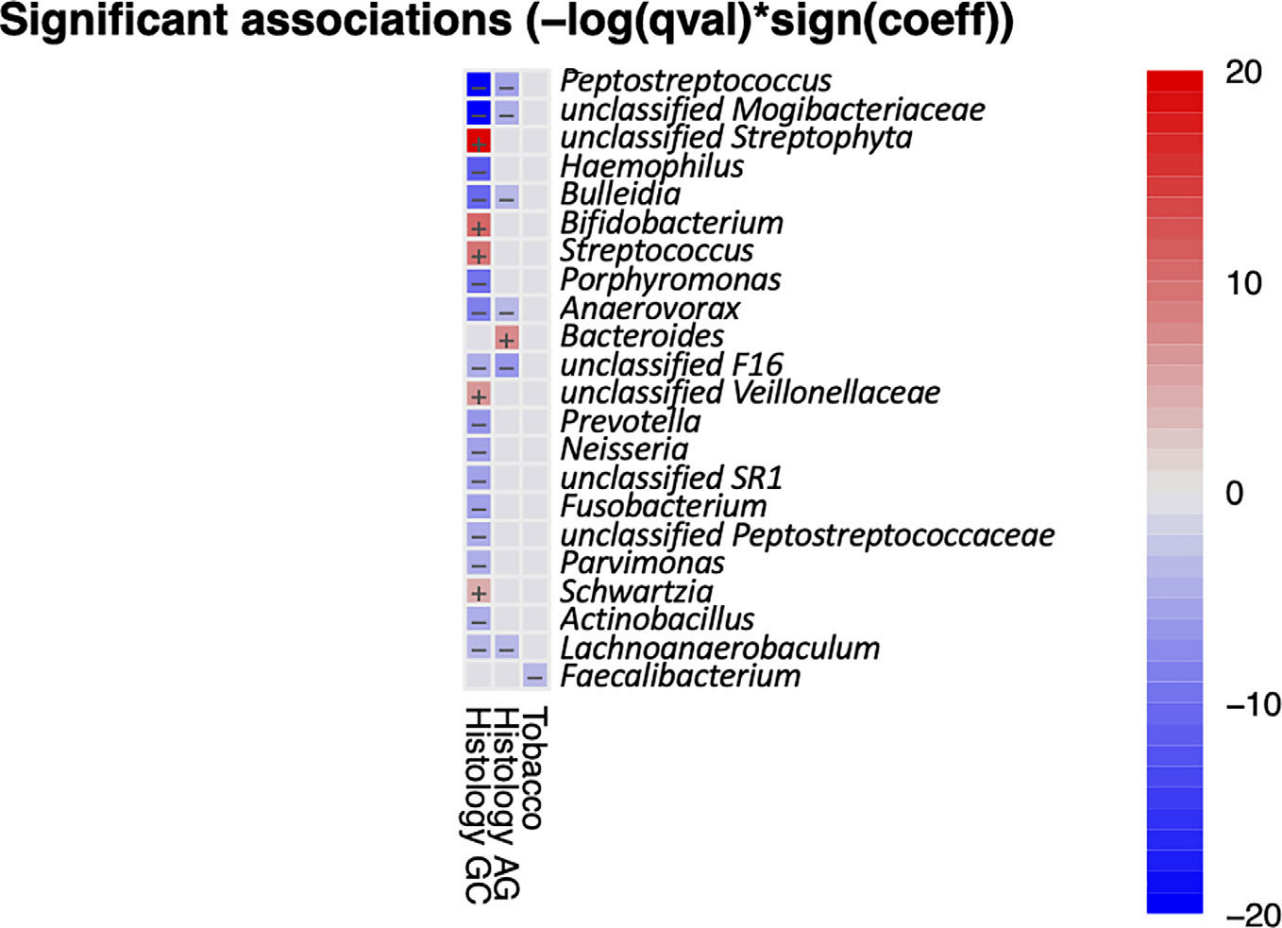
Heatmap summarizing the significant associations between oral bacteria and metadata. Color key: -log(q-value)*sign(coefficient). Cells that denote significant associations are colored (red or blue) and overlaid with a plus (+) or minus (−) sign that indicates the direction of association.

It has been shown that the composition and function of the oral microbiota are affected by lifestyle factors such as alcohol and tobacco use and health characteristics such as periodontitis, tooth status, and HP infection (Bornigen et al., 2017; Zhao et al., 2019). We used MaAsLin2 to access the multivariable association between metadata and salivary microbiota. Analysis result indicated that only *Faecalibacterium* had a significant negative correlation with tabaco usage (**Figure 3**), while no taxa were found to be significantly associated with alcohol usage, periodontosis, or HP-infection. There were 13 genera that had significant correlationships with GC, including five positive correlations (enriched in GC) and eight negative correlations(reduced in GC). Six genera were found to be negatively correlated with both AG and GC, and *Bacteroidetes* had a significant positive correlation with AG. Taken together, the results of multivariate analysis are in accordance with the results of univariate analysis.

### Salivary Microbiota Is Predictive of Stages of Gastric Carcinogenesis

**Figure 4:**
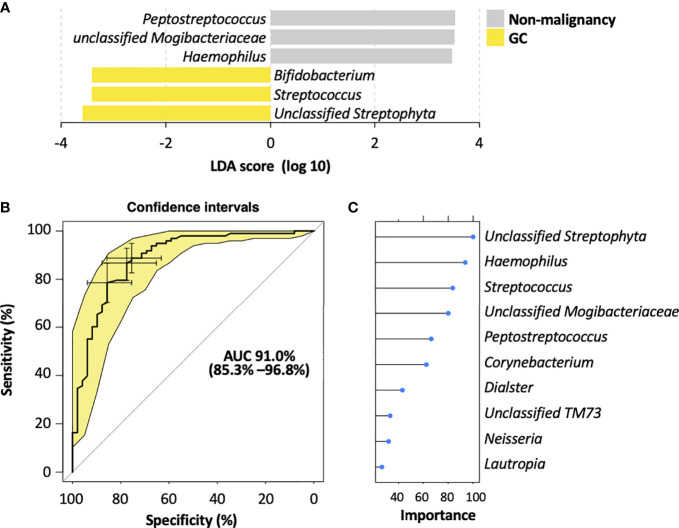
Salivary bacterial biomarkers for classifying different stages of gastric carcinogenesis. **(A)** Microbiological features of the salivary microbiota associated with different histological stages of gastric tumorigenesis. Bacteria taxa that enriched in each histological stage were determined by LEfSe with Kruskal–Wallis test *P <*0.05 and log 10 LDA score >3.4. **(B)** ROC curves analysis to evaluate the discriminatory potential of salivary bacteria in identifying GC out of pre-malignant lesions. **(C)** The top 10 bacterial genera that are most important for discriminating GC with non-malignant types. Each genus is ranked according to an importance score (mean decrease accuracy).

To identify the most relevant taxa responsible for the differences among the disease stages, we performed LEfSe analysis based on the genus (**Figure 4A**; **Figure S4A**). The representative bacterial genera in the salivary microbiota of GC patients were *unclassified Streptophyta*, *Streptococcus*, and *Bifidobacterium*; patients with AG showed increased levels of *Bacteroides* and *Haemophilus* genera; the salivary microbiota in SG patients was featured by an enrichment of *Peptostreptococcus*, *unclassified Mogibacteriaceae*, and *unclassified SR1* genera.

To further explore the potential of the salivary microbiota as diagnostic biomarkers for GC, we constructed a random forest model for identifying malignancy based on the salivary microbiota at the genus level. This model showed a high accuracy in distinguishing GC from non-malignant lesions, yielding an AUC of 0.91 (95% confidence interval 0.778–0.99) (**Figure 4B**). Moreover, the random forest modeling approach was also able to distinguish SG, AG, and GC subjects, resulting in an AUC of 0.84, 0.76, and 0.877 for SG, AG, and GC, respectively (**Supplementary Figure S4B**). Among the top 10 genera with highest contributions to the model classification performance (**Figure 4C**; **Figure S4C**), *unclassified Streptophyta* and *Streptococcus* were also identified as GC-associated microbial features by LEfSe (**Figure 4A**; **Figure S4A**), enhancing their potential in becoming biomarkers for GC diagnosis.

In addition, we performed a network analysis to visualize the commensal relationships among the salivary microbiota of the three histological types (**Figure S5**). Interestingly, we found that the correlations between *Prevotella* and other taxa dominated the negative relationships for all the three histological types, with their number reduced in AG and GC.

### Salivary Microbiome Functional Capacity in SG, AG, and GC

Analysis of PICRUSt2 revealed differentially abundant metabolic functions in the bacterial communities across the histological stages of gastric carcinogenesis (**Figure S6**). The expression of thiamin salvage II (PWY-6897), lipid IVA biosynthesis (NAGLIPASYN-PWY): CMP-3-deoxy-D-manno-octulosonate biosynthesis I (PWY-1269), Kdo transfer to lipid IVA III (PWY-6467), preQ0 biosynthesis (PWY6703), and superpathway of thiamin diphosphate biosynthesis I (THISYN-PWY) were found to be decreased from SG, through AG to GC. In contrast, pathways involved in L-isoleucine biosynthesis II (PWY-5101 and PWY-5103), L-valine biosynthesis (VALSYN-PWY), L-isoleucine biosynthesis I from threonine (ILEUSYN-PWY), superpathway of branched amino acid (BRANCHED-CHAIN-AA-SYN-PWY), pyruvate fermentation to isobutanol (PWY-7111), and UDP-N-acetyl-D-glucosamine biosynthesis I (UDPNAGSYN-PWY) were upregulated from SG through AG to GC. It is worth noting that the increased expression of biosynthesis of amino acids such as isoleucine and valine was also previously detected in the gastric cancer tissues (Jung et al., 2014; Wang et al., 2016).

## Discussion

Previous studies have shown that periodontal disease and poor oral health status were associated with increased incidence of malignant diseases (Dizdar et al., 2017; Michaud et al., 2018), pointing to a potentially oncogenic role of oral microorganisms in the development of cancer. Some species have been identified that correlate strongly with oral squamous cell carcinoma (OSCC), such as *Capnocytophaga gingivalis*, *Prevotella melaninogenica*, and *Streptococcus mitis* (Mager et al., 2005), which were suggested as diagnostic markers since they predicted 80% of cancer cases. Oral microbes are also detected in tumors distant to the oral cavity. For example, many works have shown that the oral periopathogens *Fusobacterium nucleatum* and *Porphyromonas gingivalis* are essential in the development of colorectal and pancreatic cancer, respectively (Rubinstein et al., 2013; Fan et al., 2018a). The oral microbiota was shown to reflect an inflammatory status of the stomach in patients with *H. pylori* infection (Zhao et al., 2019), and could detect GC with a high accuracy (Coker et al., 2018; Wu et al., 2018). In this study, we demonstrated that the salivary microbiota could identify GC among patients with non-malignant gastric diseases including SG and AG, yielding a high accuracy (AUC of 91%). With a cohort consisting of 37 GC patients and 13 healthy individuals, a previous study also showed a high sensitivity rate (AUC of 97%) of using oral microbiota screening gastric cancer (Sun et al., 2018), further enhancing the diagnostic potential of the oral bacteria for gastric malignancy. The microbiome is exclusive to the individual and influenced by lifestyle and phenotypic and genotypic determinants. For example, alcohol consumption and tobacco usage have been shown to influence the oral microbiome composition (Wu et al., 2016; Fan et al., 2018b). Therefore, lifestyle should be considered as confounding factors when identifying diagnostic microbial markers from the oral microbiome. Multivariate analysis method revealed that enrichment of Faecalibacterium was negatively associated with smoking, and no significant correlation was found between salivary bacteria and alcohol or HP-infection. Thus, the potential biomarkers identified based on our data seemed not affected by the recorded lifestyle and HP infection.

Our data showed that alpha diversity of the salivary microbiota was similar among patients with different gastric histological types, which was consistent with another study (Kageyama et al., 2019). One previous study found that the microbial diversity of saliva and dental plaque significantly increased in GC patients (Sun et al., 2018), whereas another one suggested that the microbiota diversity significantly reduced in the tongue coating of GC patients (Cui et al., 2019). Taken together, the microbial diversity of oral microbiota seems not strongly associated with the development of GC.

Data from our recent study (Wang et al., 2020) as well as others (Dicksved et al., 2009; Castano-Rodriguez et al., 2017; Chen et al., 2019) showed that commensals of the oral cavity including *Fusobacterium*, *Peptostreptococcus*, *Prevotella*, *Streptococcus*, and *Veillonella* were found to have higher relative abundances in the gastric mucosa of GC patients. Notably, these genera are also commensals of oral cavity, but their translocation and expansion may be involved in the onset and development of multiple diseases including cancers. One possible mechanism of oral microbiota participating carcinogenesis is enrichment of pro-inflammatory oral bacterial species. For example, *Streptococcus bovis* has been shown to promote the development of colon cancer by enhancing the inflammation (Abdulamir et al., 2011). We observed that *Streptococcus* genus was enriched in the saliva microbiota of GC patients, which agrees with the findings in a recent study (Sun et al., 2018). Interestingly, an enrichment of *Streptococcus* spp. was also reported across several types of cancer such as colorectal adenocarcinomas (Abdulamir et al., 2011). Taken together, these results indicate a potential of some strains of *Streptococcus* being involved in gastric carcinogenesis. In addition, *Corynebacterium* genus was also found to be enriched in the saliva of GC patients, which was in line with (Wu et al., 2018) a higher level *Corynebacterium;* this genus was found higher in the tongue coating microbiota community of GC patients than that of the healthy controls. Species of *Corynebacterium* are widely distributed in the microbiota of human skin, and most of them are innocuous while some species are known to cause infection such as *C. diphtheria*. In recent years, they have been increasingly reported as emerging opportunistic pathogens in immunocompromised patients with cancer, hematologic malignancy, and critical condition (Chen et al., 2012). Thus, a higher level of *Corynebacterium* spp., which appeared in the oral cavity, may reflect immune deficiency in cancer patients. Altogether, an enrichment of proinflammatory bacteria in the oral cavity is likely an import factor contributing to the development and progression of GC.

Several bacterial taxa were found reduced in the salivary microbiota of GC patients, including *Bulleidia*, *Fusobacterium*, *Haemophilus*, *Lachnoanaerobaculum*, *Neisseria, Parvimonas, Peptostreptococcus*, *Porphyromonas*, and *Prevotella*. Intriguingly, a decreased carriage of *Bulleidia* was also captured in the oral cavity of patients with esophageal squamous cell carcinoma (Chen et al., 2015). Moreover, some taxa of these genera were found to be enriched in tumor and stool samples of colorectal cancer patients, such as *Fusobacterium nucleatum, Parvimonas* micra, *Porphyromonas asaccharolytica*, and *Peptostreptococcus stomatis*, *Prevotella intermedia* (Ternes et al., 2020). And we recently found a higher load of *Fusobacterium* in the gastric mucosa of GC patients compared to SG (Wang et al., 2020). In the present study, a low level of these genera was observed in the oral cavity of GC patients as compared with SG and/or AG. In fact, bacterial abundance is majorly regulated by nutrient availability and antimicrobial signals specific to their environmental conditions. Thus, albeit these bacteria colonize and expanded in the tumor site (such as gut of patients with colorectal cancer), they may not overgrow in their original localization such as the oral cavity.

Network analysis revealed that *Prevotella* was negatively correlated with a variety of oral bacteria in the oral cavity of all three histological stages, and the number of its negative relationships decreased in AG and GC groups. In the GC patients, the abundance of *Prevotella* in the salivary cavity was lower than that of SG and AG groups, which is opposite to the findings in Sun et al.’s study (Sun et al., 2018). This discrepancy at the genus level may be explained by increasing the phylogenetic resolution *via* metagenomic sequencing and identifying the specific species/strains that related to gastric cancer.

We previously found that patients with intraepithelial neoplasia had higher relative abundances of *Haemophilus parainfluenzae* and *Nitrospirae* family in the gastric mucosa, which decreased in that of GC patients (Wang et al., 2020). Both *Haemophilus* and *Nitrospirae* are nitrate-reducing bacteria, which convert nitrate to nitrite, and also to nitric oxide (NO), which can be absorbed through the blood vessels in the oral cavity or through being swallowed into the gastrointestinal system. Accumulation of N-nitroso compounds in the gastrointestinal tract is likely to increase the risk of carcinogenesis (Forsythe and Cole, 1987; Bryan et al., 2012). Thus, the decreased abundances of *Haemophilus* in the salivary microbiota may contribute to the formation of gastric tumor.

Functional analysis based on the PICRUSt2-predicted pathways suggested that metabolic functions of salivary microbiota changed along with the disease progression in the stomach. In particular, pathways involved in isoleucine and valine biosynthesis were highly expressed by the salivary microbiota in GC patients compared to the non-malignant stages. Interestingly, an upregulation of amino acids including isoleucine and valine was also detected in human gastric tumor tissues (Jung et al., 2014; Wang et al., 2016). Higher levels of most amino acids and their primary derivatives in gastric tumor tissues were thought to be related to two main sources: the degradation of extracellular matrix by matrix metalloproteinases and the autophagic degradation of intracellular proteins (Hirayama et al., 2009). The production of amino acid from microbes in the oral cavity and gastrointestinal tract has not been quantified and deserves further investigation in terms of proliferation and survival of gastric cancer cells.

There were several limitations in this study. Firstly, we didn’t collect samples from healthy individuals as control. Secondly, amplicon sequencing of 16S rRNA has limited resolution in determining the bacterial species or strains, and it is therefore difficult to access the functions of specific bacteria involved in gastric cancer development and progression. In addition, the 16S rRNA gene V1–V3 region has been shown to provide superior taxonomic resolution for the bacterial microbiota of the human oral and respiratory tracts compared to the V3–V4 region (Zheng et al., 2015; Escapa et al., 2018). Thus, some other oral taxa with diagnostic potential for gastric cancer might not have been detected in the present data. Finally, independent clinical cohorts from multiple centers are required to evaluate the diagnostic value of the identified GC-associated saliva bacteria.

## Conclusions

We demonstrated, with a large cohort, that the salivary microbiota can be used to predict GC as well as its non-malignant stages. The contributions of the oral microbiota in the pathogenesis of GC include an accumulation of proinflammatory bacteria and a decline in those reducing carcinogenic N-nitroso compounds.

## Data Availability Statement

The data presented in the study are deposited in European Nucleotide Archive (https://www.ebi.ac.uk/ena/submit/sra/#studies), accession number PRJEB42657.

## Ethics Statement

The studies involving human participants were reviewed and approved by the ethics committee of the Chinese PLA General Hospital (No. S2016-057-02). The patients/participants provided their written informed consent to participate in this study.

## Author Contributions

YY conceived the study and revised the manuscript. KH, LW, BY, ZW, XZ, LP, JY, and GS performed the subject’s enrolment and sample collection. XG and KH analyzed clinical and sequencing data. KH and XG wrote this manuscript. All authors contributed to the article and approved the submitted version.

## Conflict of Interest

The authors declare that the research was conducted in the absence of any commercial or financial relationships that could be construed as a potential conflict of interest.

## Supplementary Material

Supplementary Table 1 Differences in the salivary microbiota compositions among patients at different progressive histological stages of gastric tumorigenesis.

Supplementary Figure 1 The salivary microbiota biodiversity in AG patients with and without intestinal metaplasia. The alpha diversity of salivary microbiota was measure at the ASV level by using **(A)** Shannon index, **(B)** Chao1. Comparison between intra-group and inter-group community distances by **(C)** unweighted UniFrac distance matrix and **(D)** weighted UniFrac distance matrix revealed no significant difference between the microbiota compositions between AG patients with and without intestinal metaplasia. *P* value was calculated by Anosim comparing the intra-group distances with between-group distances.

Supplementary Figure 2 The salivary microbiota biodiversity in GC patients with different histological grades. The alpha diversity of salivary microbiota was measure at the ASV level by using **(A)** Shannon index, and **(B)** Chao1 index. Comparison between intra-group and inter-group community distances by **(C)** unweighted UniFrac distance matrix and **(D)** weighted UniFrac distance matrix revealed no significant difference between the microbiota compositions among patients with well differentiated (W), moderately differentiated (M), and poorly differentiated(P) gastric tumor. *P* value was calculated by Anosim comparing the intra-group distances with between-group distances.

Supplementary Figure 3 The salivary microbiota compositions in patients at different histological stages of gastric tumorigenesis. The relative abundances of most abundant **(A)** phyla and **(B)** genera detected in the salivary microbiota in SG, AG, and GC patients.

Supplementary Figure 4 Microbiological features of the salivary microbiota associated with different histological stages of gastric tumorigenesis. **(A)** Bacteria taxa that enriched in each histological stage were determined by LEfSe with Kruskal–Wallis test *P*<0.05 and log 10 LDA score>3.4. **(B)** ROC curves analysis to evaluate the discriminatory potential of salivary bacteria in identifying GC out of pre-malignant lesions. **(C)**The top 10 bacterial genera that are most important for discriminating between SG, AG, and GC. Each genus is ranked according to an importance score (mean decrease accuracy).

Supplementary Figure 5 Network analyses reveal commensal relationships among the salivary bacteria. Spearman correlation network analyses showing the commensal relationships among the top 30 most abundant genera in the salivary microbiota of **(A)** superficial gastritis, **(B)** atrophic gastritis, and **(C)** gastric cancer. Taxa are represented as nodes, taxa abundance as node size, and are colored based on their belonging phylum. Edges represent significant correlations (Holm-corrected *P* < 0.05) among these taxa. Red and blue edges represent positive and negative correlations, respectively.

Supplementary Figure 6 Functional changes in the salivary microbiota are associated with the progression of gastric carcinoma. PICRUSt2 predicted metabolic pathways that significantly different in the salivary microbiota of patients at different progressive histological stages of gastric tumorigenesis. Significance was determined by using Kruskal–Wallis rank sum test with BH-adjusted *P* < 0.05.

Supplementary File 1 ASVs obtained from Naive Bayes classifier on the 16S rRNA sequencing of V3–V4 hypervariable regions.

Supplementary Code 1 R code and taxa abundance table used for constructing the random forest model and generating the AUC ROC curve.

## References

[B1] Aas J. A. Paster B. J. Stokes L. N. Olsen I. Dewhirst F. E. (2005). Defining the normal bacterial flora of the oral cavity. J. Clin. Microbiol. 43 (11), 5721–5732. 10.1128/JCM.43.11.5721-5732.2005 16272510 PMC1287824

[B2] Abdulamir A. S. Hafidh R. R. Abu Bakar F. (2011). The association of Streptococcus bovis/gallolyticus with colorectal tumors: the nature and the underlying mechanisms of its etiological role. J. Exp. Clin. Cancer Res. 30, 11. 10.1186/1756-9966-30-11 21247505 PMC3032743

[B3] Ahn J. Chen C. Y. Hayes R. B. (2012). Oral microbiome and oral and gastrointestinal cancer risk. Cancer Causes Control 23 (3), 399–404. 10.1007/s10552-011-9892-7 22271008 PMC3767140

[B4] Bolyen E. Rideout J. R. Dillon M. R. Bokulich N. A. Abnet C. C. Al-Ghalith G. A.. (2019). Reproducible, interactive, scalable and extensible microbiome data science using QIIME 2. Nat. Biotechnol. 37 (8), 852–857. 10.1038/s41587-019-0209-9 31341288 PMC7015180

[B5] Bornigen D. Ren B. Pickard R. Li J. Ozer E. Hartmann E. M.. (2017). Alterations in oral bacterial communities are associated with risk factors for oral and oropharyngeal cancer. Sci. Rep. 7 (1), 17686. 10.1038/s41598-017-17795-z 29247187 PMC5732161

[B6] Bray F. Ferlay J. Soerjomataram I. Siegel R. L. Torre L. A. Jemal A. (2018). Global cancer statistics 2018: GLOBOCAN estimates of incidence and mortality worldwide for 36 cancers in 185 countries. CA Cancer J. Clin. 68 (6), 394–424. 10.3322/caac.21492 30207593

[B7] Bryan N. S. Alexander D. D. Coughlin J. R. Milkowski A. L. Boffetta P. (2012). Ingested nitrate and nitrite and stomach cancer risk: an updated review. Food Chem. Toxicol. 50 (10), 3646–3665. 10.1016/j.fct.2012.07.062 22889895

[B8] Castano-Rodriguez N. Goh K. L. Fock K. M. Mitchell H. M. Kaakoush N. O. (2017). Dysbiosis of the microbiome in gastric carcinogenesis. Sci. Rep. 7 (1), 15957. 10.1038/s41598-017-16289-2 29162924 PMC5698432

[B9] Chen F. L. Hsueh P. R. Teng S. O. Ou T. Y. Lee W. S. (2012). Corynebacterium striatum bacteremia associated with central venous catheter infection. J. Microbiol. Immunol. Infect. 45 (3), 255–258. 10.1016/j.jmii.2011.09.016 22154992

[B10] Chen X. Winckler B. Lu M. Cheng H. Yuan Z. Yang Y.. (2015). Oral Microbiota and Risk for Esophageal Squamous Cell Carcinoma in a High-Risk Area of China. PloS One 10 (12), e0143603. 10.1371/journal.pone.0143603 26641451 PMC4671675

[B11] Chen X. H. Wang A. Chu A. N. Gong Y. H. Yuan Y. (2019). Mucosa-Associated Microbiota in Gastric Cancer Tissues Compared With Non-cancer Tissues. Front. Microbiol. 10, 1261. 10.3389/fmicb.2019.01261 31231345 PMC6560205

[B12] Coker O. O. Dai Z. Nie Y. Zhao G. Cao L. Nakatsu G.. (2018). Mucosal microbiome dysbiosis in gastric carcinogenesis. Gut 67 (6), 1024–1032. 10.1136/gutjnl-2017-314281 28765474 PMC5969346

[B13] Cui J. Cui H. Yang M. Du S. Li J. Li Y.. (2019). Tongue coating microbiome as a potential biomarker for gastritis including precancerous cascade. Protein Cell 10 (7), 496–509. 10.1007/s13238-018-0596-6 30478535 PMC6588651

[B14] Dicksved J. Lindberg M. Rosenquist M. Enroth H. Jansson J. K. Engstrand L. (2009). Molecular characterization of the stomach microbiota in patients with gastric cancer and in controls. J. Med. Microbiol. 58 (Pt 4), 509–516. 10.1099/jmm.0.007302-0 19273648

[B15] Dizdar O. Hayran M. Guven D. C. Yılmaz T. B. Taheri S. Akman A. C.. (2017). Increased cancer risk in patients with periodontitis. Curr. Med. Res. Opin. 33 (12), 2195–2200. 10.1080/03007995.2017.1354829 28699803

[B16] Douglas G. M. Maffei V. J. Zaneveld J. R. Yurgel S. N. Brown J. R. Taylor C. M.. (2020). PICRUSt2 for prediction of metagenome functions. Nat. Biotechnol. 38 (6), 685–688. 10.1038/s41587-020-0548-6 32483366 PMC7365738

[B17] Engstrand L. Lindberg M. (2013). Helicobacter pylori and the gastric microbiota. Best Pract. Res. Clin. Gastroenterol. 27 (1), 39–45. 10.1016/j.bpg.2013.03.016 23768551

[B18] Escapa I. F. Chen T. Huang Y. Gajare P. Dewhirst F. E. Lemon K. P. (2018). New Insights into Human Nostril Microbiome from the Expanded Human Oral Microbiome Database (eHOMD): a Resource for the Microbiome of the Human Aerodigestive Tract. mSystems 3 (6), e00187-18. 10.1128/mSystems.00187-18 PMC6280432 30534599

[B19] Fan X. Alekseyenko A. V. Wu J. Peters B. A. Jacobs E. J. Gapstur S. M.. (2018a). Human oral microbiome and prospective risk for pancreatic cancer: a population-based nested case-control study. Gut 67 (1), 120–127. 10.1136/gutjnl-2016-312580 27742762 PMC5607064

[B20] Fan X. Peters B. A. Jacobs E. J. Gapstur S. M. Purdue M. P. Freedman N. D.. (2018b). Drinking alcohol is associated with variation in the human oral microbiome in a large study of American adults. Microbiome 6 (1), 59. 10.1186/s40168-018-0448-x 29685174 PMC5914044

[B21] Flemer B. Warren R. D. Barrett M. P. Cisek K. Das A. Jeffery I. B.. (2018). The oral microbiota in colorectal cancer is distinctive and predictive. Gut 67 (8), 1454–1463. 10.1136/gutjnl-2017-314814 28988196 PMC6204958

[B22] Forsythe S. J. Cole J. A. (1987). Nitrite accumulation during anaerobic nitrate reduction by binary suspensions of bacteria isolated from the achlorhydric stomach. J. Gen. Microbiol. 133 (7), 1845–1849. 10.1099/00221287-133-7-1845 3117970

[B23] Gao L. Xu T. Huang G. Jiang S. Gu Y. Chen F. (2018). Oral microbiomes: more and more importance in oral cavity and whole body. Protein Cell 9 (5), 488–500. 10.1007/s13238-018-0548-1 29736705 PMC5960472

[B24] Goral V. (2016). Etiopathogenesis of Gastric Cancer. Asian Pac. J. Cancer Prev. 17 (6), 2745–2750. 27356684

[B25] Han Y. W. Wang X. (2013). Mobile microbiome: oral bacteria in extra-oral infections and inflammation. J. Dental Res. 92 (6), 485–491. 10.1177/0022034513487559 PMC3654760 23625375

[B26] He J. Li Y. Cao Y. Xue J. Zhou X. (2015). The oral microbiome diversity and its relation to human diseases. Folia Microbiol. 60 (1), 69–80. 10.1007/s12223-014-0342-2 25147055

[B27] Hirayama A. Kami K. Sugimoto M. Sugawara M. Toki N. Onozuka H.. (2009). Quantitative metabolome profiling of colon and stomach cancer microenvironment by capillary electrophoresis time-of-flight mass spectrometry. Cancer Res. 69 (11), 4918–4925. 10.1158/0008-5472.CAN-08-4806 19458066

[B28] Jean-François F. (2011). [WHO Classification of digestive tumors: the fourth edition]. Ann. Pathol. 31 (5 Suppl), S27–S31. 10.1016/j.annpat.2011.1008.1001-1031 22054452

[B29] Jung J. Jung Y. Bang E. J. Cho S. I. Jang Y. J. Kwak J. M.. (2014). Noninvasive diagnosis and evaluation of curative surgery for gastric cancer by using NMR-based metabolomic profiling. Ann. Surg. Oncol. 21 (Suppl 4), S736–S742. 10.1245/s10434-014-3886-0 25092158

[B30] Kageyama S. Takeshita T. Takeuchi K. Asakawa M. Matsumi R. Furuta M.. (2019). Characteristics of the Salivary Microbiota in Patients With Various Digestive Tract Cancers. Front. Microbiol. 10, 1780. 10.3389/fmicb.2019.01780 31428073 PMC6688131

[B31] Kauppila J. H. Selander K. S. (2014). Toll-like receptors in esophageal cancer. Front. Immunol. 5, 200. 10.3389/fimmu.2014.00200 24847326 PMC4019875

[B32] Mager D. L. Haffajee A. D. Devlin P. M. Norris C. M. Posner M. R. Goodson J. M. (2005). The salivary microbiota as a diagnostic indicator of oral cancer: a descriptive, non-randomized study of cancer-free and oral squamous cell carcinoma subjects. J. Transl. Med. 3, 27. 10.1186/1479-5876-3-27 15987522 PMC1226180

[B33] Massarrat S. Stolte M. (2014). Development of gastric cancer and its prevention. Arch. Iran. Med. 17 (7), 514–520. 24979566

[B34] Michaud D. S. Izard J. (2014). Microbiota, oral microbiome, and pancreatic cancer. Cancer J. 20 (3), 203–206. 10.1097/PPO.0000000000000046 24855008 PMC4160879

[B35] Michaud D. S. Lu J. Peacock-Villada A. Y. Barber J. R. Joshu C. E. Prizment A. E.. (2018). Periodontal Disease Assessed Using Clinical Dental Measurements and Cancer Risk in the ARIC Study. J. Natl. Cancer Inst. 110 (8), 843–854. 10.1093/jnci/djx278 29342298 PMC6093423

[B36] Peek R.M. Jr. Crabtree J. E. (2006). Helicobacter infection and gastric neoplasia. J. Pathol. 208 (2), 233–248. 10.1002/path.1868 16362989

[B37] Peters B. A. Wu J. Pei Z. Yang L. Purdue M. P. Freedman N. D.. (2017). Oral Microbiome Composition Reflects Prospective Risk for Esophageal Cancers. Cancer Res. 77 (23), 6777–6787. 10.1158/0008-5472.CAN-17-1296 29196415 PMC5726431

[B38] Plummer M. Franceschi S. Vignat J. Forman D. de Martel C. (2015). Global burden of gastric cancer attributable to Helicobacter pylori. Int. J. Cancer 136 (2), 487–490. 10.1002/ijc.28999 24889903

[B39] Polk D. B. Peek R. M. Jr (2010). Helicobacter pylori: gastric cancer and beyond. Nat. Rev. Cancer 10 (6), 403–414. 10.1038/nrc2857 20495574 PMC2957472

[B40] Rubinstein M. R. Wang X. Liu W. Hao Y. Cai G. Han Y. W. (2013). Fusobacterium nucleatum promotes colorectal carcinogenesis by modulating E-cadherin/beta-catenin signaling via its FadA adhesin. Cell Host Microbe 14 (2), 195–206. 10.1016/j.chom.2013.07.012 23954158 PMC3770529

[B41] Salazar C. R. Francois F. Li Y. Corby P. Hays R. Leung C.. (2012). Association between oral health and gastric precancerous lesions. Carcinogenesis 33 (2), 399–403. 10.1093/carcin/bgr284 22139442 PMC3384024

[B42] Segata N. Izard J. Waldron L. Gevers D. Miropolsky L. Garrett W. S.. (2011). Metagenomic biomarker discovery and explanation. Genome Biol. 12 (6), R60. 10.1186/gb-2011-12-6-r60 21702898 PMC3218848

[B43] Shi Y. C. Wang Z. K. Yang Y. S. (2019). Biobank Branch China Medicinal Biotech Association, the Digestive Biobanking Committee China Association of Medical Equipment, the Gut Microbiome Committee China Society of Gastroenterology. (2019). Consensus on standard biobanking of gut microbiota. J. Dig. Dis. 20 (3), 114–121. 10.1111/1751-2980.12705 30677249

[B44] Seymour R. A. (2010). Is oral health a risk for malignant disease? Dental Update 37 (5), 279–283. 10.12968/denu.2010.37.5.279 20669705

[B45] Sun J.-H. Li X.-L. Yin J. Li Y.-H. Hou B.-X. Zhang Z. (2018). A screening method for gastric cancer by oral microbiome detection. Oncol. Rep. 39 (5), 2217–2224. 10.3892/or.2018.6286 29498406

[B46] Sung J. J. Y. Coker O. O. Chu E. Szeto C. H. Luk S. T. Y. Lau H. C. H.. (2020). Gastric microbes associated with gastric inflammation, atrophy and intestinal metaplasia 1 year after Helicobacter pylori eradication. Gut 69 (9), 1572–1580. 10.1136/gutjnl-2019-319826 31974133 PMC7456733

[B47] Ternes D. Karta J. Tsenkova M. Wilmes P. Haan S. Letellier E. (2020). Microbiome in Colorectal Cancer: How to Get from Meta-omics to Mechanism? Trends Microbiol. 28 (5), 401–423. 10.1016/j.tim.2020.01.001 32298617

[B48] Wade W. G. (2013). The oral microbiome in health and disease. Pharmacol. Res. 69 (1), 137–143. 10.1016/j.phrs.2012.11.006 23201354

[B49] Wang H. Zhang H. Deng P. Liu C. Li D. Jie H.. (2016). Tissue metabolic profiling of human gastric cancer assessed by (1)H NMR. BMC Cancer 16, 371. 10.1186/s12885-016-2356-4 27356757 PMC4928316

[B50] Wang Z. Gao X. Zeng R. Wu Q. Sun H. Wu W.. (2020). Changes of the Gastric Mucosal Microbiome Associated With Histological Stages of Gastric Carcinogenesis. Front. Microbiol. 11, 997. 10.3389/fmicb.2020.00997 32547510 PMC7272699

[B51] Warren J. R. Marshall B. (1983). Unidentified curved bacilli on gastric epithelium in active chronic gastritis. Lancet (London England) 1 (8336), 1273–1275. 6134060

[B52] Watabe K. Nishi M. Miyake H. Hirata K. (1998). Lifestyle and gastric cancer: a case-control study. Oncol. Rep. 5 (5), 1191–1194. 10.3892/or.5.5.1191 9683833

[B53] Whitmore S. E. Lamont R. J. (2014). Oral bacteria and cancer. PloS Pathog. 10 (3), e1003933–e1003933. 10.1371/journal.ppat.1003933 24676390 PMC3968118

[B54] Wu J. Peters B. A. Dominianni C. Zhang Y. Pei Z. Yang L.. (2016). Cigarette smoking and the oral microbiome in a large study of American adults. ISME J. 10 (10), 2435–2446. 10.1038/ismej.2016.37 27015003 PMC5030690

[B55] Wu J. Xu S. Xiang C. Cao Q. Li Q. Huang J.. (2018). Tongue Coating Microbiota Community and Risk Effect on Gastric Cancer. J. Cancer 9 (21), 4039–4048. 10.7150/jca.25280 30410609 PMC6218773

[B56] Yang L. Chaudhary N. Baghdadi J. Pei Z. (2014). Microbiome in reflux disorders and esophageal adenocarcinoma. Cancer J. 20 (3), 207–210. 10.1097/PPO.0000000000000044 24855009 PMC4120752

[B57] Zhao Y. Gao X. Guo J. Yu D. Xiao Y. Wang H.. (2019). Helicobacter pylori infection alters gastric and tongue coating microbial communities. Helicobacter 24 (2), e12567. 10.1111/hel.12567 30734438 PMC6593728

[B58] Zheng W. Tsompana M. Ruscitto A. Sharma A. Genco R. Sun Y.. (2015). An accurate and efficient experimental approach for characterization of the complex oral microbiota. Microbiome 3, 48. 10.1186/s40168-015-0110-9 26437933 PMC4593206

