# Supplementary figures and images for: Salivary Microbiota for Gastric Cancer Prediction: An Exploratory Study
Source: Front Cell Infect Microbiol. 2021 Mar 10;11:640309. doi: 10.3389/fcimb.2021.640309 (PMC7988213; doi:10.3389/fcimb.2021.640309)

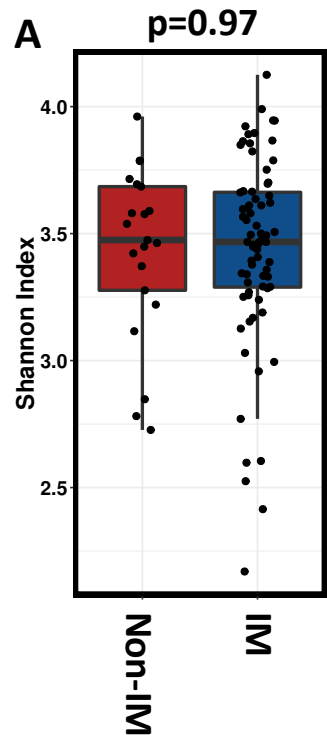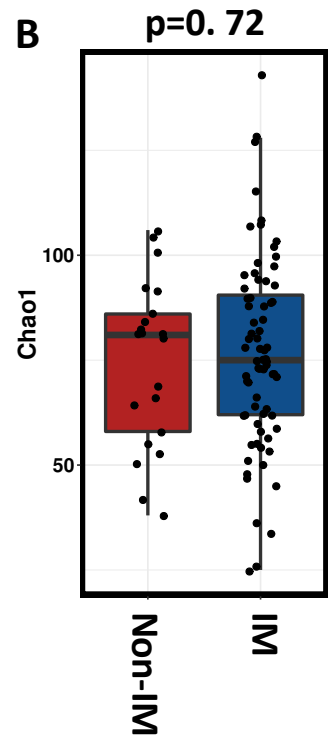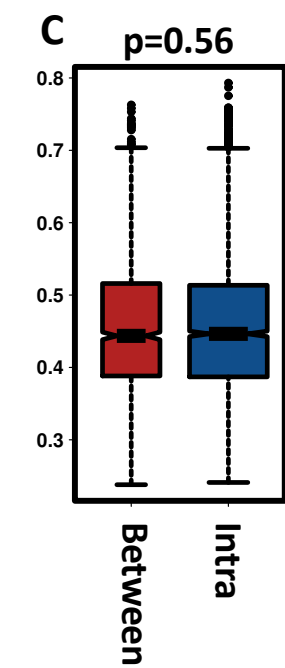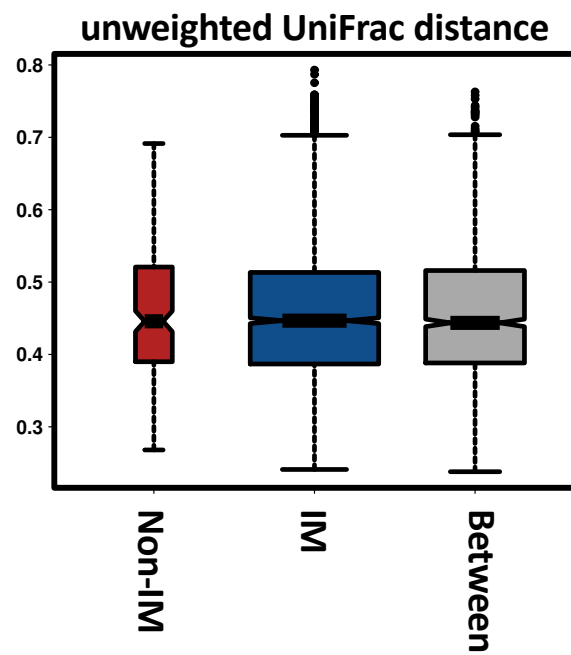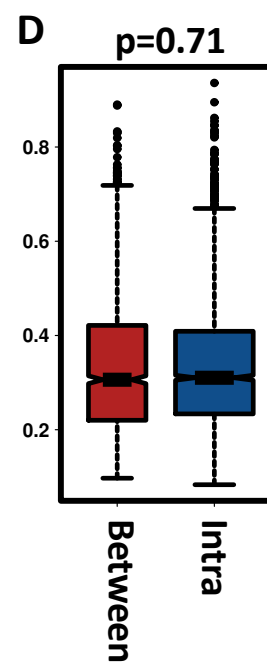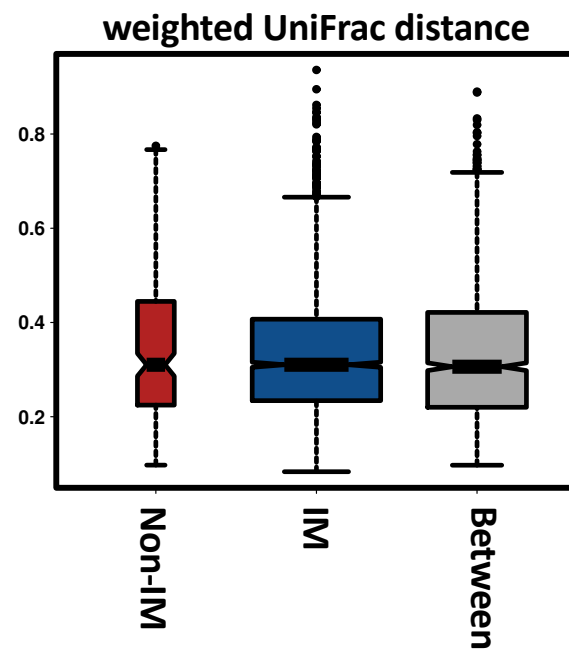

Supplement: Supplementary Figure 1 — The salivary microbiota biodiversity in AG patients with and without intestinal metaplasia. The alpha diversity of salivary microbiota was measure at the ASV level by using (A) Shannon index, (B) Chao1. Comparison between intra-group and inter-group community distances by (C) unweighted UniFrac distance matrix and (D) weighted UniFrac distance matrix revealed no significant difference between the microbiota compositions between AG patients with and without intestinal metaplasia. P value was calculated by Anosim comparing the intra-group distances with between-group distances. [file Image_1.pdf]

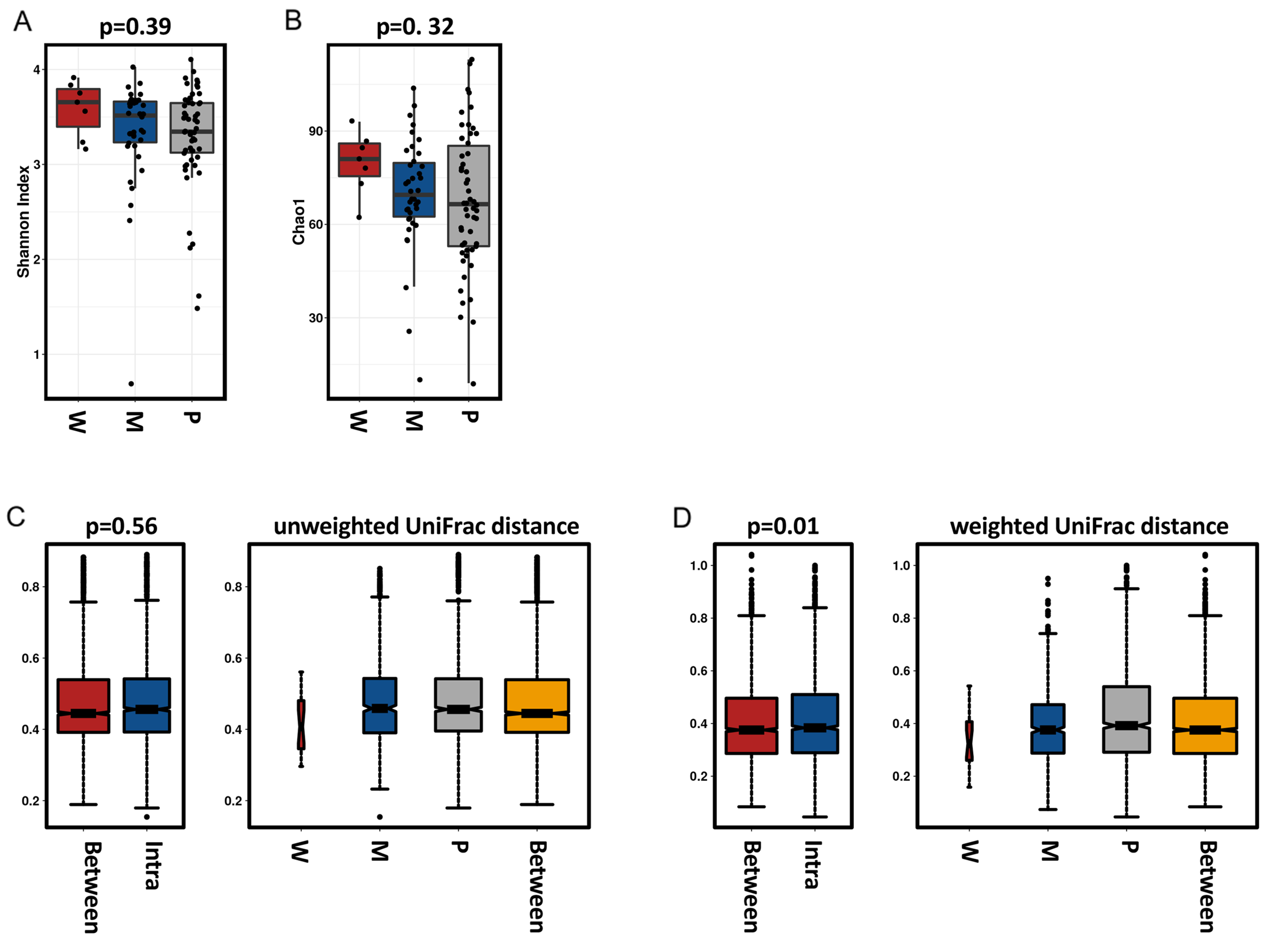

Supplement: Supplementary Figure 2 — The salivary microbiota biodiversity in GC patients with different histological grades. The alpha diversity of salivary microbiota was measure at the ASV level by using (A) Shannon index, and (B) Chao1 index. Comparison between intra-group and inter-group community distances by (C) unweighted UniFrac distance matrix and (D) weighted UniFrac distance matrix revealed no significant difference between the microbiota compositions among patients with well differentiated (W), moderately differentiated (M), and poorly differentiated(P) gastric tumor. P value was calculated by Anosim comparing the intra-group distances with between-group distances. [file Image_2.tiff]

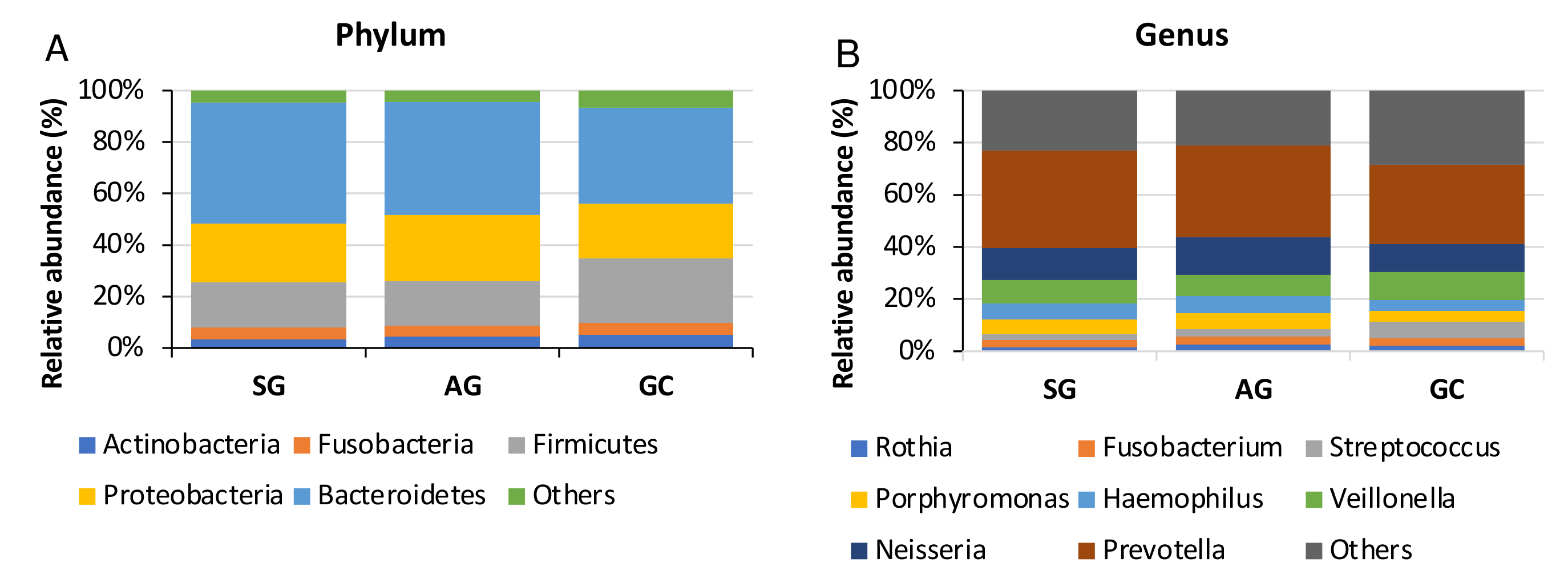

Supplement: Supplementary Figure 3 — The salivary microbiota compositions in patients at different histological stages of gastric tumorigenesis. The relative abundances of most abundant (A) phyla and (B) genera detected in the salivary microbiota in SG, AG, and GC patients. [file Image_3.tiff]

**A**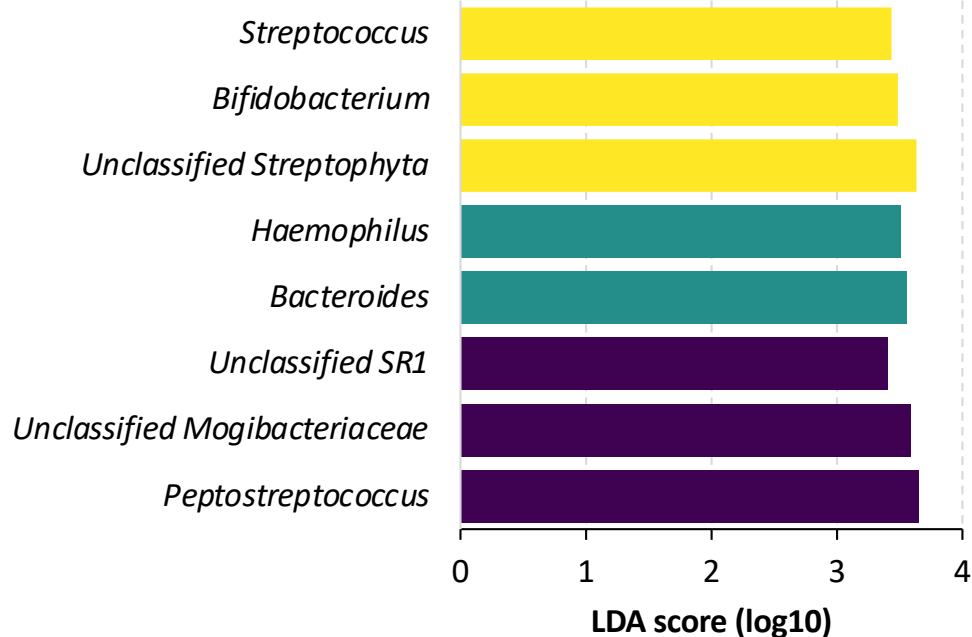**B**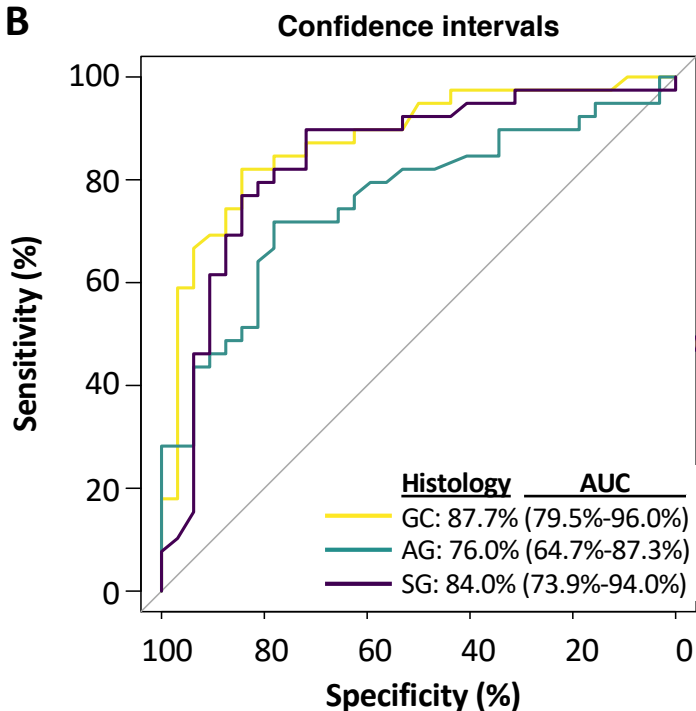**C**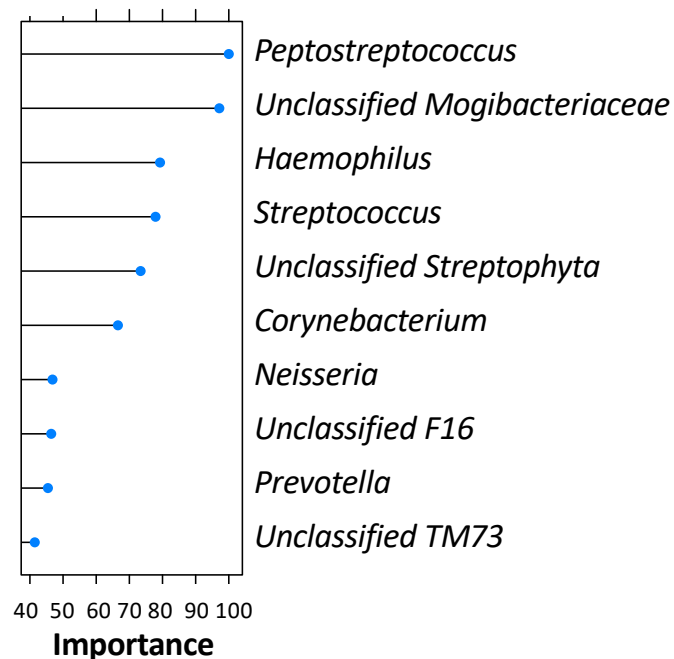

Supplement: Supplementary Figure 4 — Microbiological features of the salivary microbiota associated with different histological stages of gastric tumorigenesis. (A) Bacteria taxa that enriched in each histological stage were determined by LEfSe with Kruskal–Wallis test P<0.05 and log 10 LDA score>3.4. (B) ROC curves analysis to evaluate the discriminatory potential of salivary bacteria in identifying GC out of pre-malignant lesions. (C)The top 10 bacterial genera that are most important for discriminating between SG, AG, and GC. Each genus is ranked according to an importance score (mean decrease accuracy). [file Image_4.pdf]
