# Supplementary material for: Salivary Microbiota for Gastric Cancer Prediction: An Exploratory Study
Source: Front Cell Infect Microbiol. 2021 Mar 10;11:640309. doi: 10.3389/fcimb.2021.640309 (PMC7988213; doi:10.3389/fcimb.2021.640309)
Supplement: Supplementary Figure 5 — Network analyses reveal commensal relationships among the salivary bacteria. Spearman correlation network analyses showing the commensal relationships among the top 30 most abundant genera in the salivary microbiota of (A) superficial gastritis, (B) atrophic gastritis, and (C) gastric cancer. Taxa are represented as nodes, taxa abundance as node size, and are colored based on their belonging phylum. Edges represent significant correlations (Holm-corrected P < 0.05) among these taxa. Red and blue edges represent positive and negative correlations, respectively. [file Image_5.pdf]

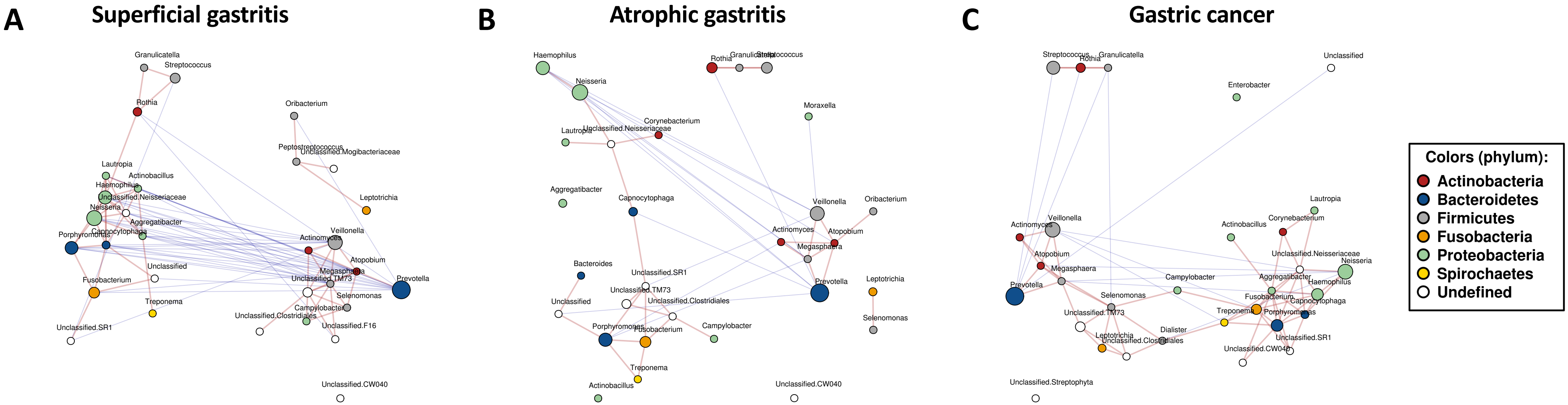

A
Superficial gastritis
Granulicatella
Streptococcus
Rothia
Oribacterium
Peptostreptococcus
Unclassified.Mogibacteriaceae
Lautropia
Actinobacillus
Haemophilus
Unclassified.Neisseriaceae
Neisseria
Aggregatibacter
Capnocytophaga
Porphyromonas
Leptotrichia
Veillonella
Actinomyces
Atopobium
Megasphaera
Unclassified
Unclassified.TM73
Prevotella
Fusobacterium
Selenomonas
Treponema
Campylobacter
Unclassified.Clostridiales
Unclassified.F16
Unclassified.SR1
Unclassified.CW040
B
Atrophic gastritis
Haemophilus
Rothia
Granulicatella
Streptococcus
Neisseria
Moraxella
Corynebacterium
Lautropia
Unclassified.Neisseriaceae
Aggregatibacter
Capnocytophaga
Veillonella
Oribacterium
Actinomyces
Atopobium
Megasphaera
Bacteroides
Unclassified.SR1
Prevotella
Leptotrichia
Unclassified.TM73
Unclassified
Unclassified.Clostridiales
Selenomonas
Porphyromonas
Fusobacterium
Campylobacter
Treponema
Actinobacillus
Unclassified.CW040
C
Gastric cancer
Streptococcus
Rothia
Granulicatella
Unclassified
Enterobacter
Lautropia
Actinomyces
Veillonella
Actinobacillus
Corynebacterium
Atopobium
Unclassified.Neisseriaceae
Neisseria
Megasphaera
Campylobacter
Aggregatibacter
Prevotella
Fusobacterium
Haemophilus
Selenomonas
Capnocytophaga
Unclassified.TM73
Treponema
Porphyromonas
Dialister
Leptotrichia
Unclassified.Clostridiales
Unclassified.SR1
Unclassified.CW040
Unclassified.Streptophyta
Colors (phylum):
Actinobacteria
Bacteroidetes
Firmicutes
Fusobacteria
Proteobacteria
Spirochaetes
Undefined
