# Supplementary material for: Salivary Microbiota for Gastric Cancer Prediction: An Exploratory Study
Source: Front Cell Infect Microbiol. 2021 Mar 10;11:640309. doi: 10.3389/fcimb.2021.640309 (PMC7988213; doi:10.3389/fcimb.2021.640309)
Supplement: Supplementary Figure 6 — Functional changes in the salivary microbiota are associated with the progression of gastric carcinoma. PICRUSt2 predicted metabolic pathways that significantly different in the salivary microbiota of patients at different progressive histological stages of gastric tumorigenesis. Significance was determined by using Kruskal–Wallis rank sum test with BH-adjusted P < 0.05. [file Image_6.pdf]

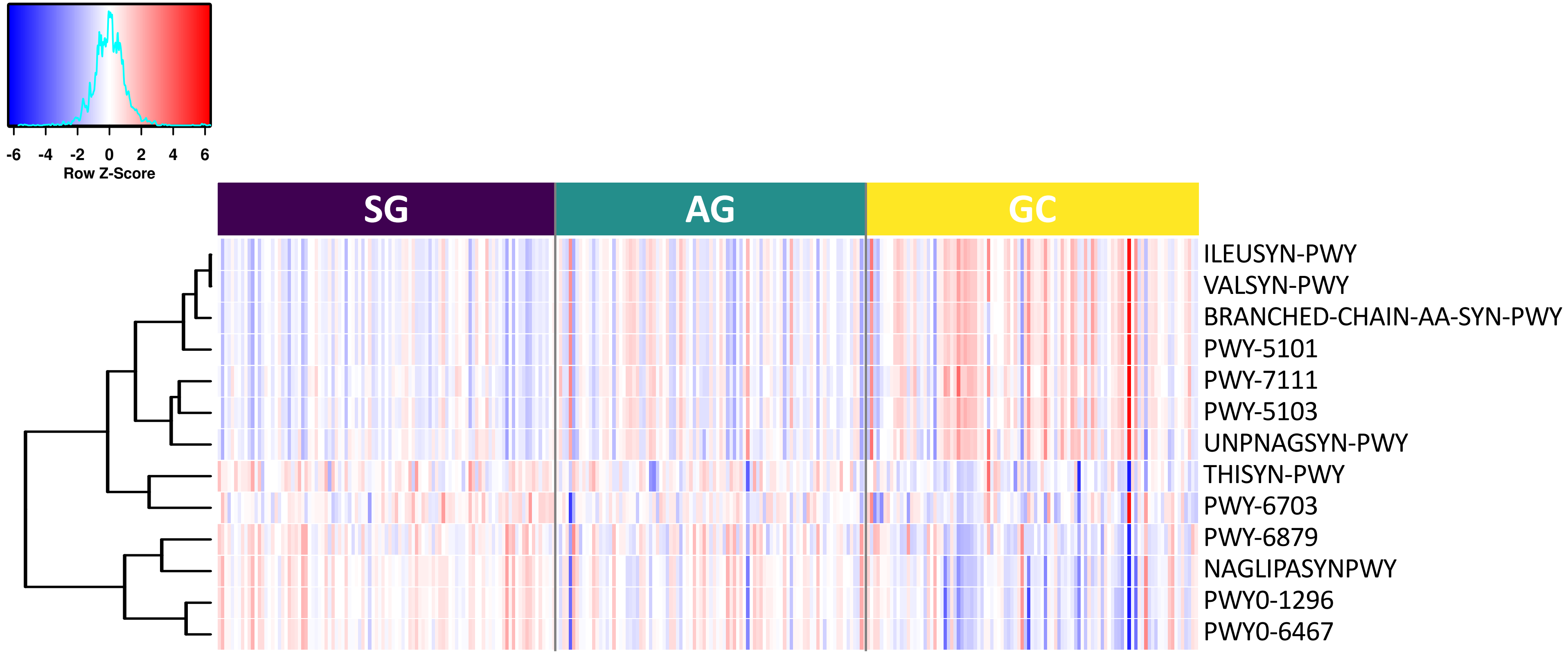

ILEUSYN-PWY: L-isoleucine biosynthesis I (from threonine)
VALSYN-PWY: L-valine biosynthesis
BRANCHED-CHAIN-AA-SYN-PWY: superpathway of branched amino acid
PWY-5101: L-isoleucine biosynthesis II
PWY-5103: L-isoleucine biosynthesis III
PWY-7111: pyruvate fermentation to isobutanol (engineered)
UDPNAGSYN-PWY: UDP-N-acetyl-D-glucosamine biosynthesis I

THISYN-PWY: superpathway of thiamin diphosphate biosynthesis I
PWY6703: preQ0 biosynthesis
PWY-6897: thiamin salvage II
NAGLIPASYN-PWY: lipid IVA biosynthesis
PWY-1269: CMP-3-deoxy-D-manno-octulosonate biosynthesis I
PWY-6467: Kdo transfer to lipid IVA III (Chlamydia)
